# Saracatinib, a Src Tyrosine Kinase Inhibitor, as a Disease Modifier in the Rat DFP Model: Sex Differences, Neurobehavior, Gliosis, Neurodegeneration, and Nitro-Oxidative Stress

**DOI:** 10.3390/antiox11010061

**Published:** 2021-12-28

**Authors:** Meghan Gage, Marson Putra, Logan Wachter, Kylie Dishman, Megan Gard, Crystal Gomez-Estrada, Thimmasettappa Thippeswamy

**Affiliations:** Department of Biomedical Sciences and Interdepartmental Neuroscience Program, Iowa State University, Ames, IA 50011, USA; mcgage@iastate.edu (M.G.); mrputra@iastate.edu (M.P.); lwachter@iastate.edu (L.W.); kdishman@iastate.edu (K.D.); mgard@iastate.edu (M.G.); cgomez21@stu.psm.edu (C.G.-E.)

**Keywords:** diisopropylfluorophosphate, saracatinib, Src family kinase, neuroinflammation, neurodegeneration, nitro-oxidative stressors, epilepsy, behavioral comorbidities

## Abstract

Diisopropylfluorophosphate (DFP), an organophosphate nerve agent (OPNA), exposure causes status epilepticus (SE) and epileptogenesis. In this study, we tested the protective effects of saracatinib (AZD0530), a Src kinase inhibitor, in mixed-sex or male-only Sprague Dawley rats exposed to 4–5 mg/kg DFP followed by 2 mg/kg atropine and 25 mg/kg 2-pralidoxime. Midazolam (3 mg/kg) was given to the mixed-sex cohort (1 h post-DFP) and male-only cohort (~30 min post-DFP). Saracatinib (20 mg/kg, oral, daily for 7 days) or vehicle was given two hours later and euthanized eight days or ten weeks post-DFP. Brain immunohistochemistry (IHC) showed increased microgliosis, astrogliosis, and neurodegeneration in DFP-treated animals. In the 10-week post-DFP male-only group, there were no significant differences between groups in the novel object recognition, Morris water maze, rotarod, or forced swim test. Brain IHC revealed significant mitigation by saracatinib in contrast to vehicle-treated DFP animals in microgliosis, astrogliosis, neurodegeneration, and nitro-oxidative stressors, such as inducible nitric oxide synthase, GP91^phox^, and 3-Nitrotyrosine. These findings suggest the protective effects of saracatinib on brain pathology seem to depend on the initial SE severity. Further studies on dose optimization, including extended treatment regimen depending on the SE severity, are required to determine its disease-modifying potential in OPNA models.

## 1. Introduction

Organophosphate nerve agents (OPNAs) pose significant threats to both military and civilian populations. These agents have previously been used in chemical warfare scenarios, such as during the Iraq–Iran war and later in terrorist attacks, which suggests that they may be used in the future [1,2,3,4,5,6]. OPNAs include a wide array of molecules, including G-series agents such as Soman (GD), Sarin (GB), and cyclosarin (GF), V-series agents such as VX and VG, GV series, and Novichok [7,8]. These NAs cause cholinergic toxicity through the irreversible inhibition of acetylcholinesterase (AChE), accumulation of acetylcholine (ACh) at the synapses, and overactivation of ACh receptors [9,10,11]. This leads to a wide variety of symptoms, such as bronchoconstriction, salivation, lacrimation, bradycardia, gastrointestinal distress, and convulsions [12,13,14]. OPNA poisoning, depending on the concentrations, can lead to *status epilepticus* (SE), a period of continuous convulsive seizures, which can initiate epileptogenesis, the development of epilepsy due to permanent brain injury in the long term [15,16,17]. Importantly, epilepsy is associated with spontaneously recurring seizures (SRS), gliosis, neurodegeneration, and oxidative stress, which can lead to behavioral comorbidities such as cognitive deficits, increased prevalence of depression and anxiety, and, in some instances, motor deficits [18,19,20,21,22,23,24,25]. Many research groups have used animal models to investigate the mechanisms of epileptogenesis following the initiation of SE by OPNAs. Diisopropylflourophosphate (DFP), an OP, is often used as a surrogate for other more potent OPNAs such as soman. Our laboratory and others have shown the development of SE and epilepsy following administration of DFP [26,27,28,29,30,31,32].

OPNA toxicity is typically combatted with the administration of anticholinergic drugs (antidotes) such as atropine (inhibition of ACh receptors) and oximes (reverses the aging of AChE) if administered immediately [14,33,34,35]. Importantly, these antidotes have only peripheral effects and do not cross the blood–brain barrier (BBB), thus having minimal effects in reversing brain toxicity [36,37]. Benzodiazepines, such as diazepam (DZP) or midazolam (MDZ), are effective in terminating behavioral SE, but when the administration is delayed, they are ineffective in preventing epileptogenesis [30,38]. Military personnel are pretreated with pyridostigmine bromide, but recent animal studies have shown no improvement in mortality or the development of SE [39,40,41]. Additional therapeutic targets need to be investigated for effective mitigation of the development of epilepsy and associated comorbidities following OPNA-induced toxicity.

Although ion channels have typically been the target for antiseizure and antiepileptic drugs discovery, neuroinflammation, a hallmark of epilepsy, has more recently become a therapeutic target for disease modification [24,42,43]. In this study, we explore the therapeutic potential of saracatinib (SAR, also known as AZD0530), a Src family tyrosine kinase (SFKs) inhibitor, following epileptic insult by the OPNA, DFP. SFKs are non-receptor tyrosine kinases that are involved in a wide variety of biological processes. SFKs such as Fyn and Src are expressed in the brain and mediate synaptic plasticity, long-term potentiation, cell proliferation, and neuronal modulation [44,45,46,47]. We have recently reviewed the roles of SFKs in neurological diseases [48]. In neurons, Fyn can phosphorylate metabotropic glutamate receptors (mGluR1) as well as the GluN2A and GluN2B units of N-methyl D-aspartate receptors (NMDAR) [49,50,51]. Our laboratory and others have shown that Fyn can also phosphorylate Tau, which can lead to translocation of the Fyn–Tau complex to the membrane to activate NMDARs [52,53,54]. Additionally, there is also evidence that Fyn could negatively modulate GABAergic transmission, suggesting its role in seizure generation [55,56]. Fyn phosphorylates PKCδ lead to the activation of the mitogen-activated protein kinase (MAPK) pathway and translocation of nuclear factor-kappa light chain enhancer (NFκB) to the nucleus [57,58]. NFκB induces the transcription of a variety of molecules, including proinflammatory cytokines and inducers of oxidative stress [59,60,61]. This would suggest that inhibitors of SFKs could be beneficial in the mitigation of disease via antioxidant pathways. Fyn’s role in both neuronal excitability and neuroinflammation demonstrate its utility as a possible target for the modulation of epileptogenesis [48].

The SFK inhibitor, SAR, is a small molecule that has been tested in clinical trials for cancers such as ovarian cancer and small-cell lung cancer [62,63]. More recently, it has been tested in animal models of neurological diseases such as Alzheimer’s (AD) and Parkinson’s disease (PD) [64,65,66]. It has also been tested in clinical trials of AD, though with limited efficacy [67]. Possibly, SAR administration may be more beneficial if started early in the disease onset and progression. Importantly, we have shown that SAR has the ability to cross the BBB in mice and rats as we were able to detect it in the hippocampus 5–8 h after oral administration [57]. In this study, mice pretreated with SAR had a reduction in SE severity following the challenge by kainic acid (KA). When SAR was administered to rats after the induction of SE by KA, there was a significant reduction in the occurrence of SRS [68]. Another study using pilocarpine to initiate SE in mice showed that SAR administration led to a reduction in epileptogenic spikes [69]. This suggests that SAR may also be a useful disease modifier following SE induced by an organophosphate, DFP.

We recently tested the short-term effects of SAR in animals that had about 20 min of convulsive seizures after DFP intoxication [70]. These animals did not develop SRS during the treatment period and had significantly reduced neurodegeneration compared to the VEH-treated group when tested 24 h after the last day of SAR treatment (i.e., 8 days post-DFP) [70]. Notably, the SAR-treated animals had significant weight loss compared to VEH controls. In that study, we administered 25 mg/kg SAR, beginning 4 h after DFP intoxication, twice daily at 12 h intervals during the first three days, followed by a single dose per day for the next four days [70]. In this study, we tested 20 mg/kg per day for 7 days to minimize toxicity and increase the efficacy of the drug. We also tested the short-term (8 days, in both sexes) and the long-term (10 weeks, males only) impact of this dosing regimen. We hypothesize that SAR administration with this dosing regimen for a week will mitigate DFP-induced brain pathology and morbidity.

## 2. Materials and Methods

### 2.1. Animal Source, Care and Ethics

Age-matched (7–8 weeks) males and females or males only Sprague-Dawley rats were purchased from Charles River (Wilmington, MA, USA). Animals were allowed at least 72 h of acclimation before experiments began. The number of male and female animals sacrificed at each timepoint is outlined in Table 1.

Procedures were approved by the Iowa State University Animal Care and Use Committee (IACUC-21-109). Animals were provided ad libitum access to food and water by animal care staff at the Iowa State University Laboratory of Animal Resources. Animals were single-housed, and males and females were kept in separate rooms with temperatures between 19–22 °C. Animals were randomized for DFP or phosphate-buffered saline (PBS) injection. At the end of the experiment, animals were euthanized with 100 mg/kg pentobarbital sodium (in euthanasia solution, i.p.) as per the American Veterinary Medical Associations Guidelines for the Euthanasia of Animals. All procedures complied with the ARRIVE guidelines [71].

### 2.2. Chemicals and Reagents

DFP (Sigma-Aldrich, St. Louis, MO, USA; >97% purity by GC-MS) was prepared fresh in cold 0.1 M PBS 5 min prior to administration and kept on ice throughout the experiment. Atropine sulfate (ATS, Thermo Fisher Scientific, Waltham, MA, USA) and pralidoxime (2-PAM, Sigma Aldrich) were also prepared in saline just prior to administration and kept at room temperature. MDZ and euthanasia solution were purchased from the Iowa State University Lloyd Veterinary Medical Center Hospital Pharmacy. Paraformaldehyde (PFA) was purchased from Acros Organics and prepared fresh (4%) in PBS prior to perfusion. Gelatin, used for tissue embedding, consisted of the following: 15% type A porcine gelatin, 7.5% sucrose, 0.1% sodium azide in PBS. Citric acid buffer for antigen retrieval consisted of 10 mM citric acid and 0.05% Tween 20, at pH 6.0. For immunohistochemistry (IHC), blocking buffer consisted of 10% donkey serum and 0.2% Triton X in 0.1 M PBS. Antibodies were diluted in 2.5% donkey serum, 0.25% sodium azide, 0.1% Triton X in 0.1 M PBS. Streptavidin was diluted in 0.1 M PBS. Antibodies source and concentrations used are listed in Table S1. For Western blotting, radioimmunoprecipitation (RIPA) buffer and protease and phosphatase inhibitor (PPI) were purchased from Thermo Fisher Scientific. Antibodies (for Western blotting) were diluted in 1:1 blocking buffer (Licor) and 0.1% Tween 20 to reduce background. SAR was kindly supplied by AstraZeneca through the Open Innovation Program and diluted in 0.5% hydroxypropylmethylcellulose and 0.1% Tween 80 (VEH). SAR was prepared once in 3–4 days and left stirring at room temperature to prevent precipitation. SAR formulation has been described in our previous publication [70].

### 2.3. Exposure to Diisopropylflourophosphate (DFP)

**Figure 1:**
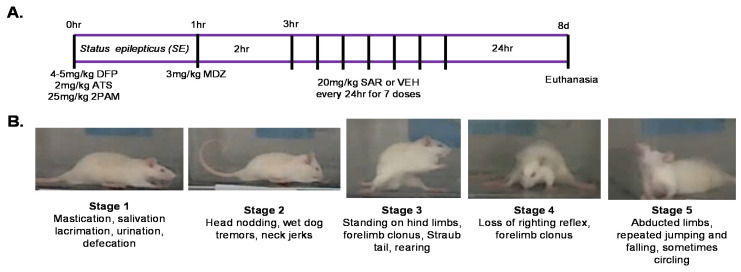
Experimental design and seizure staging for short time-point studies (day 8 post-DFP). (**A**) Male and female rats were exposed to 4 mg/kg and 5 mg/kg, diisopropylfluorophosphate (DFP), respectively, followed 1 min later by 2 mg/kg atropine sulfate (ATS) and 25 mg/kg pralidoxime (2-PAM). Most animals started to display seizure activity within 5–10 min of DFP intoxication (status epilepticus-SE). One hour later, 3 mg/kg midazolam (MDZ) was administered followed two hours later by 20 mg/kg saracatinib (SAR) or vehicle (VEH). SAR or VEH was administered once a day for 7 days. 24 h after the last dose, animals were euthanized for immunohistochemical analysis. (**B**) Behavioral seizures of each stage (1–5) are described. During SE, animals were assessed in real-time for the seizure severity.

We used a total of 103 animals in this study. We had four treatment groups as listed in Table 1; PBS + VEH, PBS + SAR, DFP + VEH, and DFP + SAR. Animals were administered 4 mg/kg (males) or 5 mg/kg (females) DFP (s.c.) or PBS as a control. DFP or PBS administration was randomized. We decided to increase the dose in females due to the increased resistance of females to develop seizures following DFP in our previous publication [26]. Immediately after the administration of DFP, animals were administered 2 mg/kg ATS and 25 mg/kg pralidoxime (2 PAM, i.m.) to reduce mortality. The experimental timeline is illustrated in Figure 1A. Around 5–10 min after DFP administration, animals began displaying behavioral seizures. We ranked these seizures using a modified Racine scale in which stages 1–2 are considered non-convulsive seizures (NCS) and stages 3–5 are considered convulsive seizures (CS) [72]. Behavioral features are described and depicted in Figure 1B. Stage one seizures involve salivation, lacrimation, urination, defecation, and mastication, while stage two involves the addition of head nodding and tremors. Stage three is characterized by rearing, forelimb clonus, and Straub tail; stage four is characterized by loss of the righting reflex, while stage five seizures are characterized by circling and repeated falling. For the 8-day group, MDZ was administered one hour after DFP intoxication (*n* = 33). The number of animals (*n*) used as controls, VEH, DFP with VEH/SAR are listed in Table 1. For the 10-week group, MDZ was administered after the animals displayed 20 min of CS (*n* = 32). For animals that did not reach 20 min of CS, MDZ was administered one hour after DFP administration. Treatments were assigned in a randomized block design based on the number of minutes in CS in order to mitigate the effects of SE severity in epileptogenesis. SAR or VEH was administered two hours after MDZ in order to account for the time it would take a patient to reach the hospital in a real-world scenario. In both groups, 20 mg/kg SAR (p.o.) or VEH was administered every 24 h for 7 total doses. Animals were administered saline (1 mL, s.c.) and nutrical (Midwest Veterinary Supply) twice a day for at least the first three days until the animals gained weight. Animals were handled twice a day for the duration of the treatment period and were assessed for the occurrence of SRS (induced due to handling) and morbidity.

### 2.4. Modified Irwin Scoring for Morbidity Analysis

24 h after DFP exposure, animals were assessed using a modified version of the Irwin scale [73,74]. We evaluated a total of ten parameters; a point was given for the presence of each parameter. The parameters are as follows: ocular changes such as exophthalmos, porphyrin (bulged eyes and staining around the eyes) and lacrimation, hunched body posture, tremors, jumpiness (unprovoked or when probed by experimenter), muscle weakness (tail rigidity), aggression (actively trying to bite experimenter during handling), diarrhea, change in fur color, and piloerection. In addition, we also recorded when animals had SRS during handling for the first seven days. The animals were each handled twice a day.

### 2.5. Behavioral Evaluation

Epilepsy is associated with a variety of behavioral comorbidities. The animals were rested for 6 weeks after DFP exposure, prior to behavioral testing, to allow progression of epileptogenesis. One day rest was given between each test. Prior to experimentation, animals were left to acclimate to the testing environment. Two experimenters conducted the behavioral testing so that the handling was performed by the same person throughout the experiment. The ANY-maze video-tracking software (Stoelting Co., Wood Dale, IL, USA) was used to measure various parameters.

#### 2.5.1. Novel Object Recognition

The apparatus consisted of a square 100 cm/100 cm box. One day prior to testing, animals acclimated to and explored the empty apparatus for 10 min. Between each animal, the apparatus was sterilized with 70% ethanol to counter the influence of animal odor. On the next day, animals were placed into the apparatus for 5 min with two identical objects placed in opposite corners of the apparatus. Importantly, animals were released from a corner opposite to either object. Four hours later, animals were placed in the same apparatus for 5 min with one of the familiar objects and a novel object. The time spent with each object was measured. In order to gain a sense of long-term memory, 24 h later, we placed the animals into the apparatus with the first familiar object and a second novel object for 5 min. Importantly, animals that spend more time with the novel object are presumed to remember the old object; thus, this test measures learning and cognition [75]. We also placed a separate group of normal animals (*n* = 8, Figure S1) into the apparatus with all three objects and found that there was no statistical difference in the time the animals spent with each object (Note: these 8 animals are not included in Table 1 since they were not used in this study except for the validation purpose).

#### 2.5.2. Horizontal Bar Test

The apparatus consisted of a wooden bar (80 cm long), 60 cm above a table surface. The animal was released from one end of the bar and trained (by the experimenter pushing the animal) to walk to a safety box at the other end of the bar. On the first day, a 4 cm wide beam was used, and four consecutive trials were given. The same procedure was repeated on the second day. On the third day, the 4 trials were conducted using a 2 cm wide bar. Three hours later, the animal was then tested in the same manner on a 1 cm bar. For each trial period, the time to reach the platform, number of times the experimenter needed to push the animal, and number of footslips (foot not being placed correctly on the bar) were measured.

#### 2.5.3. Rotarod

Animals were placed in groups of four onto a rotating rod (AccuRotor 4 channel, Omnitech Electronics Inc., Columbus, OH, USA) 36 cm above a surface. Animals were placed onto the rod (7 cm diameter; initial velocity 10 rpm) and accelerated to 60 rpm over 2 min. The acquisition software, Fusion 6.2, detected and recorded the time took for an animal to fall from the rotating rod. Latency to fall was considered a measure of motor ability. Animals were given three trials a day for two days, followed by a test.

#### 2.5.4. Morris Water Maze (MWM)

The apparatus consists of a black circular tank (180 cm diameter, 60 cm high) filled with water (23–26 °C). The tank was divided into four quadrants (SE, SW, NW, NE) which each had a distinct visual cue above the water. Animals are required to find an invisible (plexiglass) platform (submerged 2–3 cm below the water) in the tank. Over time, animals learn to associate the location of the platform with visual cues. This requires spatial learning and memory, which can be impaired in epileptic rats [76]. On the first day, the platform was placed in the middle of the tank approximately an inch above the water, so that the animals could see the location of the platform. This training encourages exploration in the tank and helps the animals to learn that the platform is in the tank. Animals were given four trials with a maximum time of 120 s with inter-trial intervals of 25–30 min. On the next day, the platform was hidden under the water in one of the quadrants (target quadrant). Animals were again given four trials with a maximum of 120 s with 25–30-min inter-trial intervals. Animals were released from a different quadrant for each trial; the order of the quadrants was randomized between animals. The training protocol with the submerged platform was repeated for 5 more days. Then, 24 h after training, the platform was removed from the apparatus. Animals were released from the opposite quadrant to the target quadrant and allowed to swim for 60 s. The amount of time spent in the target quadrant indicates the impact of treatment (DFP and SAR/VEH) on memory.

#### 2.5.5. Forced Swim Test

The forced swim test is considered a measure of depression and anxiety [77]. Animals are placed into a large cylinder (54 cm tall, 28 cm diameter) filled with water (23–26 °C). Animals were placed in the water and allowed to acclimate for 2 min. For 4 min after the acclimation period, the degree of immobility was measured by ANY-maze software. Increased immobility is considered to be a marker of increased depression and anxiety.

### 2.6. Immunohistochemistry

Animals were perfused with 0.1 M PBS for 2 min, followed by 4% PFA in 0.1 M PBS for 15–20 min. Following perfusion, brains were incubated in PFA for 24 h before transferring to 25% sucrose in 0.1 M PBS. Brains were then gelatin embedded overnight prior to freezing with liquid nitrogen and cooling with isopentane before storing at −80 °C. Brains were sectioned coronally and serially at 16 μm thick so that each slide contained 4–5 sections approximately 480 μm apart [78,79]. A total of 4–5 sections were used for each immunohistochemical stain. Sections were stored at −20 °C for long-term use. For antigen retrieval, slides were immersed in citric acid buffer at 90–95 °C for 23 min and then left to cool for 30 min. Slides were mounted onto Shandon cover plates and then placed in Shandon racks and washed with PBS for an hour. Slides were incubated with blocking buffer for an hour prior to incubation with primary antibodies overnight at 4 °C. The next day, slides were cooled for 15 min before washing with PBS for an hour. Slides were then incubated with appropriate biotinylated or alexaflour conjugated secondary species antibodies for an hour. After washing with PBS for another hour, slides were then incubated with streptavinin-CY3 for an hour, followed by another hour of washing with PBS. For flourojade B (FJB) staining, slides were washed with distilled water before incubation in 0.006% potassium permanganate for 5 min. After washing with distilled water, slides were immersed in 0.0003% FJB in 0.1% acetic acid for 10 min and dehydrated with xylene. Slides were then mounted with vectashield containing DAPI or Surgipath acrytol and stored in the fridge until imaged.

Two microscopes were used to image the hippocampus (Cornu Ammonis 1 (CA1), Cornu Ammonis 3 (CA3), and dentate gyrus (DG), piriform cortex (PC), and amygdala (AMY)). For NeuN-FJB staining, we used the Axiovert 200 Zeiss inverted fluorescence microscope with the Hammamatsu camera. Other stains were imaged with the Leica DMi8 microscope with the Leica K5 camera. Image J (FIJI) was used to manually quantify IBA1, GFAP, CD68, FJB, iNOS, 3NT and GP91^phox^ positive cells; experimenters were blind to the treatment group at the time of quantification. DAPI was used in order to confirm the presence of various molecules inside of cells. We counted from at least 4 sections for each staining/animal, and when calculating the total number of cells, we considered the average between the sections.

### 2.7. Western Blotting

Following euthanasia with sodium pentobarbital, brains were dissected to extract the PC-AMY region. Following dissection, tissues were snap-frozen in liquid nitrogen and stored at −80 °C. Lysates were prepared by homogenizing the tissues in RIPA buffer (1 μg/μL) containing 0.1% protease and phosphatase inhibitors. Lysates were then sonicated and centrifuged for an hour at 10 RCF. A Bradford protein assay was used to determine the volume required for 30–60 μg/sample. Samples (containing protein and sample buffer) were added to wells of an SDS-PAGE gel (8–10%). An amount of 2Μl molecular weight marker was used in a separate well to determine the size of the proteins. SDS-PAGE was run for ~2 h at 100 Mv (4 °C). Gels were transferred (wet) onto a nitrocellulose membrane for 16 h at 4 °C, washed, and blocked for an hour. Membranes were incubated overnight in primary antibodies followed by washing in PBS containing 0.1% Tween 20 (PBST) for an hour. Appropriate IR dye-conjugated secondary antibodies were used to detect the primary antibody with incubation for an hour followed by another hour washing in PBST. A similar method was used with housekeeping control protein, β-actin (incubation for an hour with both primary and secondary antibodies). Odessey IR imaging system and Image studio Lute were used to visualize and analyze protein concentrations.

### 2.8. Experimental Design, Methodological Rigor, and Statistics

Extreme care was taken to maintain the integrity of the research. Importantly, we randomized treatment in a way that seizure severity was matched across treatment groups. Where appropriate, experimenters were blind to the treatment group. We utilized the Grubbs’ test to identify outliers and the Shapiro–Wilk test to assess normality. A linear mixed model was used to determine the effects of sex on various parameters in different brain regions. A simple linear regression model was used to determine the correlation between the markers of oxidative stress and the number of reactive microglia and astrocytes in each treatment group. Graphing and statistical procedures were performed using Graphpad Prisim 9.0. Specific statistics details can be found in the corresponding figure legends. *p*-values of <0.05 were considered statistically significant.

## 3. Results

### 3.1. Impact of DFP and SAR Treatment: SE Severity, Weight Loss, SRS, and Mortality

**Figure 2:**
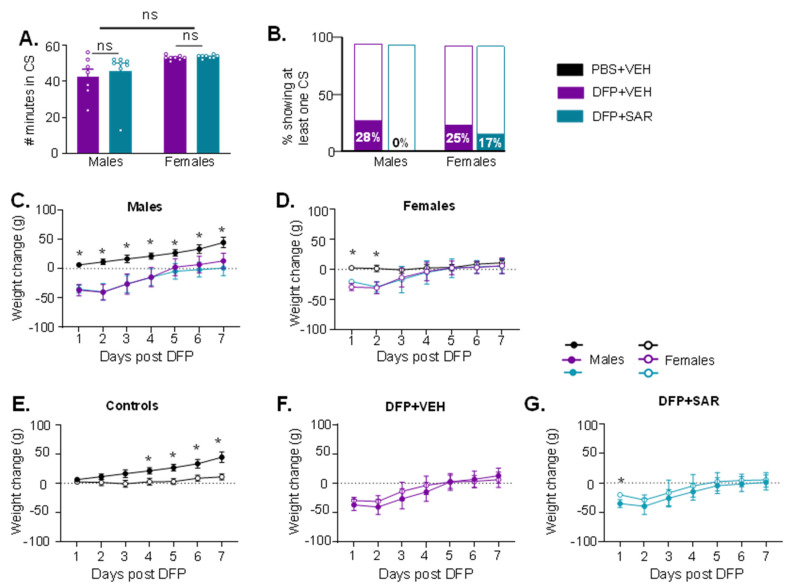
Initial seizure (SE) severity and morbidity measures during the treatment period for animals with 1 h between DFP exposure and midazolam treatment. (**A**) There was no statistical difference in the amount of time spent in CS during SE between animals treated with saracatinib (SAR) or vehicle (VEH) in either sex or between sexes (*t*-test, *n* = 6–8). (**B**) 28% of VEH treated males and 25% of VEH treated females had at least one CS when handled during the treatment period compared to none in SAR treated males but 17% in SAR treated females (Fisher’s exact). (**C**,**D**) All DFP treated males and females had significant weight loss compared to controls (*n* = 5–8, mixed measures ANOVA). (**D**) Control males (PBS + VEH) gained weight significantly faster than female controls but there was no difference in the DFP animals regardless of VEH or SAR treatment. (**E**–**G**): Weight change comparison between males and females in controls, DFP + VEH, and DFP + SAR. * *p* < 0.05, *n* = 5–8, mixed measures ANOVA.

We challenged males with 4 mg/kg and females 5 mg/kg DFP, or an equal volume of VEH, followed by 2 mg/kg ATS, 25 mg/kg 2-PAM (immediately) and MDZ (after one hour). There were no differences in the initial SE severity between animals that were used to treat with SAR or VEH or for either sex, nor were there sex differences (Figure 2A). None of the VEH-treated males or females died, while 2/10 males (2 and 6 days post-DFP) and 2/8 females (2 and 4 days post-DFP) treated with SAR died. Further, 28% of VEH-treated males (*n* = 7) and 25% of VEH-treated females (*n* = 8) had at least one CS during handling compared to none in SAR-treated males (*n* = 8) and 17% in SAR-treated females (*n* = 6) (Figure 2B). The differences were not significantly different between VEH- and SAR-treated animals. DFP intoxication led to significant weight loss for the first 3–4 days post-exposure in both males and females, and SAR treatment had no effect (Figure 2C,D). We also compared males and females in each treatment group (Figure 2E–G). Male controls gained weight significantly quicker than female controls (Figure 2E). There were no differences in weight loss between sexes for DFP-treated animals, with the exception of the first day of DFP and SAR males compared to DFP and SAR females (Figure 2G).

### 3.2. Impact of DFP Toxicity and SAR Treatment on Short-Term (8 Days) Gliosis and Neurodegeneration

We used a linear mixed model to consider treatment and the location in the brain. In order to evaluate sex differences on a more holistic basis, we pooled data from all hippocampal regions (CA1, CA3 and DG) as well as data from the PC and AMY. A linear mixed model was also used to determine the effects of sex on various parameters. Since the initial SE severity between sexes was not significantly different (Figure 2A), it is therefore appropriate to compare males and females across the various parameters.

#### 3.2.1. Microgliosis

**Figure 3:**
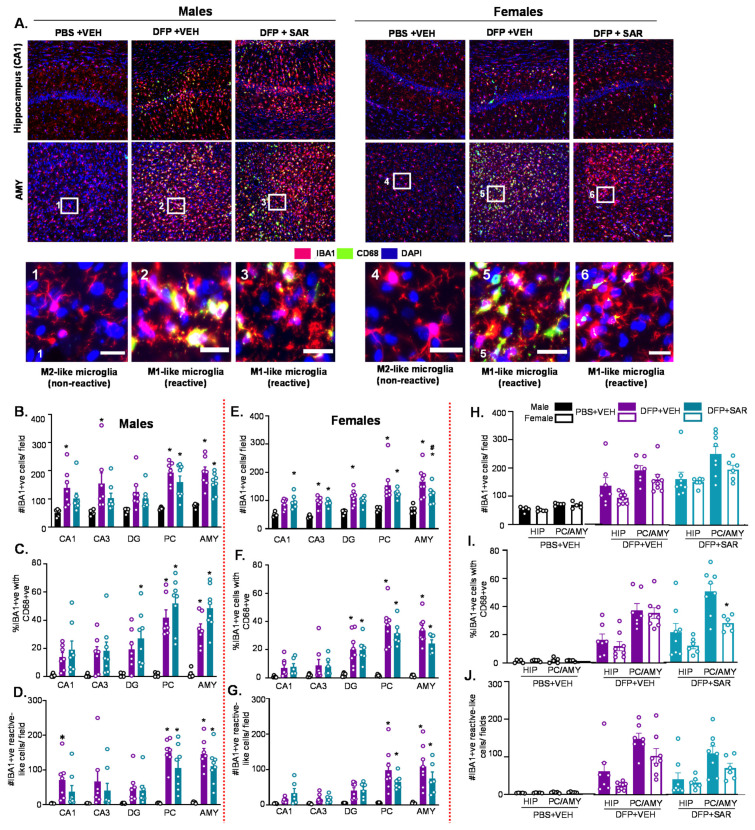
Microgliosis at day 8 post-DFP. (**A**) Representative images of IBA1 and CD68 positive cells (immunohistochemistry-IHC) from each treatment group. Scale, 50 μm for whole field (0.44 m^2^) images and 25 μm for boxed images (1–6). (**B**–**J**) Cell quantification. (**B**,**E**) Number of IBA1 positive cells. (**C**,**F**) Percent IBA1 positive cells colocalized with CD68 positive cells. (**D**,**G**) Number of IBA1 positive cells with reactive-type morphology. Reactive cells were considered to have large cell bodies and retracted processes. (**H**–**J**) Averages were pooled from the hippocampus (CA1, CA3 and DG) as well as the PC and AMY to compare sex differences for all microgliosis parameters. For all, *n* = 5–8, mixed measures ANOVA. * *p* < 0.05 compared to controls and # *p* < 0.05 compared to DFP + VEH (B-G). * *p* < 0.05 males vs females (**H**–**J**).

The number of IBA1 positive cells, the number of CD68 positive cells, and the number of IBA1 positive cells with reactive morphology (M1-like) were quantified. Reactive microglia have large cytoplasm and short processes in contrast to non-reactive microglia, which have small cell bodies and long processes [80]. Representative images of IBA1 and CD68 from CA1 of the hippocampus and the AMY are presented in Figure 3A. As expected, we found significant upregulation of IBA1 positive cells in DFP- and VEH-treated males and females compared to respective control in all regions of the brain (Figure 3B,E). This was not significantly mitigated by SAR in most regions except in the AMY in females (Figure 3E). There was also a significant upregulation of the percent of IBA1 positive cells with CD68 in DFP-treated males and females (both SAR and VEH) compared to controls in the DG, PC, and AMY (Figure 3C,F). There was also a significant upregulation of the number of IBA1 positive cells with reactive morphology in the PC and AMY (Figure 3D,G). Overall there were minimal sex differences in the number of IBA1 positive cells, percent cells with CD68, and the number of reactive-like microglia (Figure 3H–J). The only significant difference was in the percent cells with CD68, in which females had significantly less than males in the PC/AMY region in the SAR-treated group (Figure 3I).

#### 3.2.2. Astrogliosis

**Figure 4:**
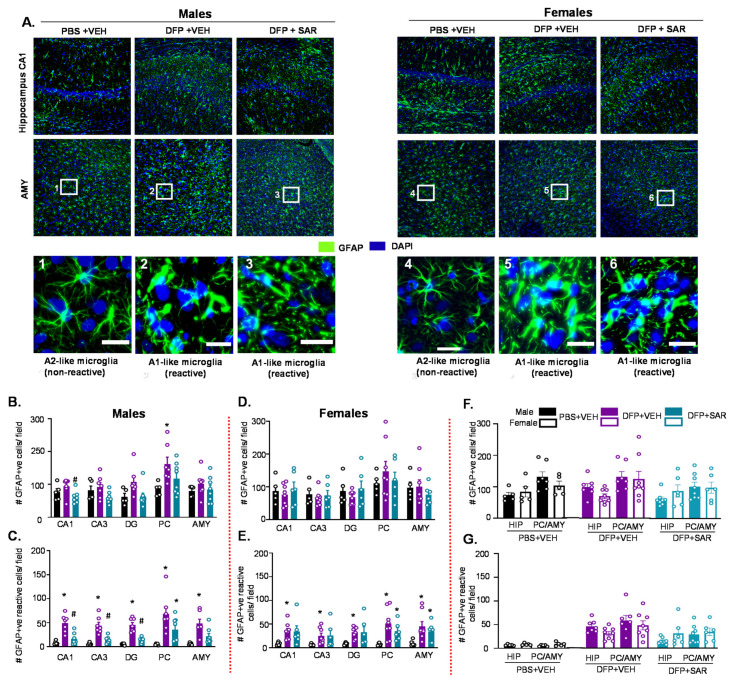
Astrogliosis at day 8 post-DFP. (**A**) Representative IHC images for GFAP positive cells from each treatment group. Scale, 50 μm for whole field (0.44 m^2^) images and 25 μm for boxed images (1–6). (**B**–**G**) Cell quantification. (**B**,**D**) Number of GFAP positive cells. (**C**,**E**) Number of GFAP positive cells with reactive-type morphology. Reactive cells were considered to have large cell bodies and retracted processes. (**F**,**G**) Averages were pooled from the hippocampus (CA1, CA3 and DG) as well as the PC and AMY to compare sex differences for all astrogliosis parameters. For all, *n* = 5–8, mixed measures ANOVA. * *p* < 0.05 compared to controls and # *p* < 0.05 compared to DFP + VEH (**B**–**E**). * *p* < 0.05 males vs females (**F**–**G**).

We counted the total number of GFAP positive cells and the number of GFAP positive cells with reactive morphology. Reactive cells had retracted processes and large cell bodies [81]. Representative images of astrogliosis in the hippocampus (CA1) and AMY from both male and female groups are shown in Figure 4A. Interestingly, DFP and SAR treatment did not lead to a significant change in the number of GFAP positive cells with the exception of DFP and VEH males in the PC (Figure 4B,D). However, in males, all regions of the brain had significant upregulation in reactive astrocytes by DFP and significant reduction by SAR treatment in the hippocampal regions (Figure 4C). In females, there was also an increase in GFAP positive cells with reactive morphology, but the mitigation by SAR was not as significant as in males (Figure 4E). There were no significant sex differences in the hippocampus or AMY/PC region for the number of GFAP positive cells or the number of GFAP positive cells with reactive-like morphology in any treatment group (Figure 4F,G).

#### 3.2.3. Neurodegeneration

**Figure 5:**
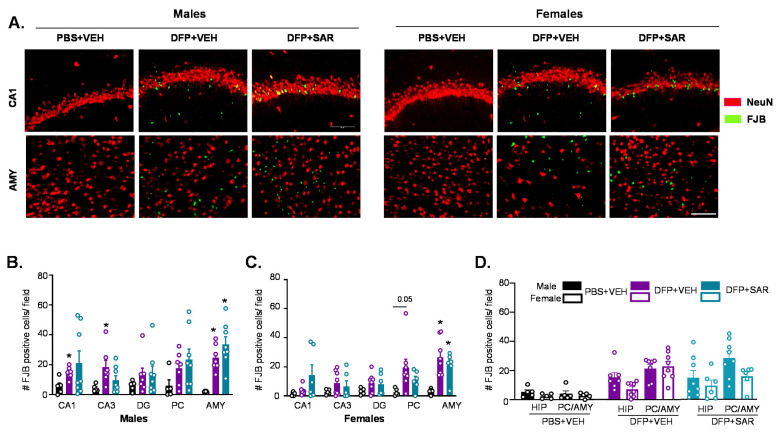
Neurodegeneration at day 8 post-DFP. (**A**) Representative IHC images for NeuN and FJB positive cells from each treatment group. Scale, 50 μm. (**B**,**C**) Number of FJB positive cells. (**D**) Averages were pooled from the hippocampus (CA1, CA3 and DG) as well as the PC and AMY to compare sex differences. For both, *n* = 5–8, mixed measures ANOVA; * *p* < 0.05 compared to controls and # *p* < 0.05 compared to DFP + VEH (**B**,**C**). * *p* < 0.05 males vs females; *p*-values indicated numerically when *p* < 0.1 (**D**). Field is 0.44 m^2^.

We found FJB positive cells that were colocalized with NeuN as well as FJB positive cells without NeuN positive staining, as reported in our previous publication [70]. We counted both colocalized cells and FJB cells without NeuN in our assessment of neurodegeneration since NeuN was expected to downregulate in some neurons in response to stress [82]. Representative images from the hippocampus (CA1) and AMY are shown in Figure 5A. DFP-treated males and females had increased FJB positive cells in all regions of the brain, though it was only significant in some regions (Figure 5B,C). This was not significantly mitigated by SAR treatment. There were no sex differences in the number of FJB positive cells for any group in any brain regions investigated (Figure 5D).

### 3.3. DFP and SAR Effects in Animals with ~20 min of Continuous CS (SE): Weight Loss, Mortality, SRS, and Morbidity

**Figure 6:**
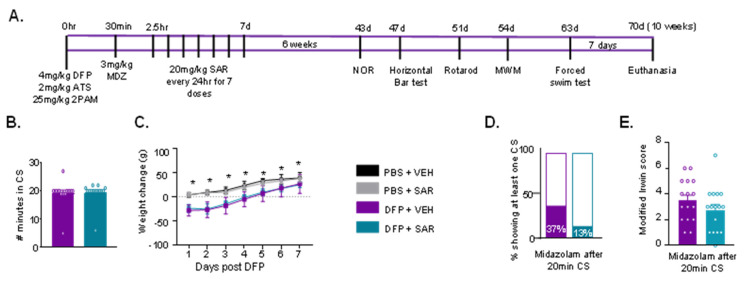
(**A**) Experimental design for long-term effects of DFP intoxication and mitigation by SAR (10 weeks post-DFP). Male rats were challenged with 4 mg/kg DFP followed by 2 mg/kg atropine sulfate (ATS) and 25 mg/kg pralidoxime (2-PAM). About 5-10 min post DFP, animals began to display convulsive seizures (CS). After 20 min of first CS midazolam (MDZ) was administered (i.e., ~30 min post-DFP). Two hours after MDZ, animals were administered 20 mg/kg saracatinib (SAR) or vehicle (VEH) once a day for 7 days. Six weeks after DFP intoxication, animals were tested for behavioral comorbidities: novel object recognition (NOR), horizontal bar test, rotarod, Morris water maze (MWM), and forced swim test. (**B**–**E**) Seizure severity and morbidity measures during the treatment period for animals with ~20 min SE. (**B**) There was no difference in the number of minutes animals spent in CS during SE between SAR and VEH treated animals (*t*-test, *n* = 15–16). (**C**) Bodyweight change over the treatment period for each treatment group (*n* = 13–16, mixed measures ANOVA). (**D**) 37% of VEH treated animals and 13% of SAR treated animals had at least one spontaneous convulsive seizure (CS) while handling during the treatment period (Fisher’s exact). (**E**) Animals were assessed for morbidity measures using the modified Irwin scale (*n* = 15–16, *t*-test). * *p* < 0.05.

Since severe SE with continuous CS lasting for >40 min seems to have caused a significant brain injury and limited mitigation by SAR (one-week treatment), we tested its efficacy in animals with ~20 min of SE. The experimental design is presented in Figure 6A.

Importantly, there was no difference in the duration of CS during SE between VEH- and SAR-treated animals (Figure 6B). None of the animals (*n* = 16) in the DFP and VEH group died, while one of the animals in the DFP and SAR group died about 24 h after MDZ (1/16). Notably, one of the PBS and VEH animals also died shortly after MDZ administration. DFP-treated animals (VEH and SAR) lost weight for the first 2–3 days post-DFP, which was significantly greater compared to controls (Figure 6C). We recorded when animals had SRS during animal handling (twice a day). Results showed that 37% of DFP and VEH animals and 13% of DFP and SAR animals had at least one SRS during the treatment period (Figure 6D, not significant). We also assessed other comorbidities in DFP-treated animals based on a modified Irwin scale at 24 h post-DFP; at the time, only one dose of SAR or VEH had been administered. SAR-treated animals had lower Irwin scores (21.4%) than VEH-treated animals, though the difference was not significant (Figure 6E).

### 3.4. Impact of DFP and SAR on Behavioral Comorbidities

Six weeks following DFP intoxication, animals were tested on a variety of behavioral apparatuses to assess learning, memory, motor ability, anxiety and depression. The order of experiments is depicted in Figure 6A.

#### 3.4.1. Impact of DFP and SAR on Learning and Memory

**Figure 7:**
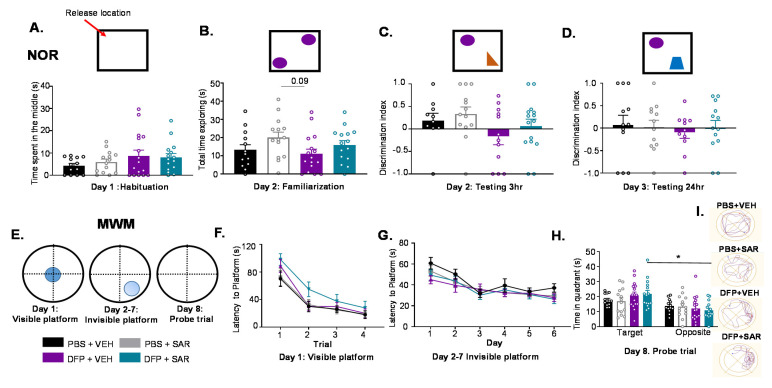
Effects of DFP and SAR or VEH on learning and memory in the novel object recognition test (NOR) and Morris water maze (MWM). (**A**–**D**) Experimental design for the NOR is illustrated in boxes. (**A**) On the first day (habituation) animals were allowed to explore the empty apparatus, a large square arena (100 cm^2^). Amount of time spent in the center of the apparatus for each treatment group is presented. (**B**) On the next day (familiarization), animals were placed into the same apparatus with two identical objects; amount of time animals spent exploring the objects is presented. (**C**) Three hours later, one of the objects was replaced with a novel object. (**D**) 24 h post-familiarization, a new novel object replaced the previously used novel object. Higher discrimination indices indicate a greater proportion of time spent with the novel object. (**E**) Experimental design for the MWM. The apparatus consisted of a large tank (60 cm high, 180 cm diameter) filled with water; several visually distinct cues lined the tank. On the first day, a visible platform was placed in the center of the tank; animals were trained to reach the platform. On days 2–7, animals were trained (4 trials a day) to find an invisible platform using the cues. On the 8th day, animals were released into the tank without a platform and time spent in the target quadrant (prior platform location) was measured (probe trial). (**F**) Time to the visible platform for four trials during the first day of training. (**G**) Time to the invisible platform on days 2–7 of training. (**H**) Time spent in the target and opposite quadrant during the probe trial. (**I**) Representative trial plots from each group. All, *n* = 13–16, ANOVA, or mixed measures ANOVA. * *p* < 0.05; *p*-values indicated numerically when *p* < 0.1.

Animals were assessed for learning and memory in the novel object recognition test. A graphical representation of the test is illustrated in top panels in Figure 7A–D. On the first day of the test, there was no significant difference between any treatment groups in the amount of time animals spent in the center of the apparatus (Figure 7A). On the second day, we measured the time the animals spent exploring either object and found no difference between the treatment groups (Figure 7B). Three hours after familiarization, animals were placed into the apparatus with the familiar object and a novel object. DFP and VEH groups had 53% lower (though not significant) discrimination indices compared to controls (treated with PBS) that were mitigated by SAR by 73%, though the differences were not significant (Figure 7C). Testing was again completed 24 h later with a different novel object to assess long-term memory. There were no significant differences between the groups, and discrimination indices were low for all groups (Figure 7D).

To further test learning and memory, animals were assessed by the MWM test. A graphical representation of the test is presented in Figure 7E. On the first day, animals were placed in the water with the platform visible at the center of the tank. Over four trials, all treatment groups found the platform faster; there was no significant difference between the groups (Figure 7F). On days 2–7, animals were tested for reaching a submerged platform in the water. There were no significant differences in the number of times animals took to find the submerged platform between any treatment groups (Figure 7G). Then, 24 h later, animals were placed in the tank for 60 s without the platform. All treatment groups spent more time in the target quadrant, though this was only significant for the DFP- and SAR-treated animals (Figure 7H). A representative trial for each group is presented in Figure 7I.

#### 3.4.2. Impact of DFP and SAR on Anxiety, Depression and Motor Activity

**Figure 8:**
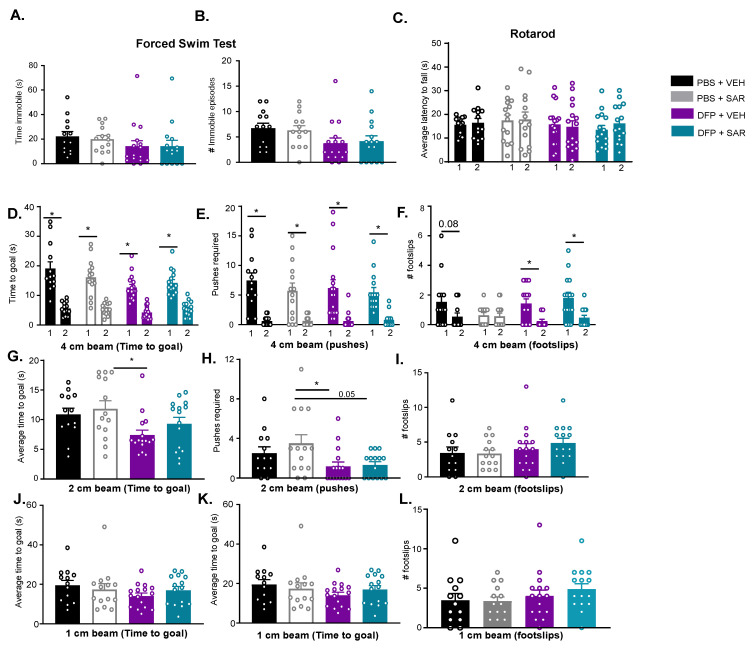
Effects of DFP and SAR or vehicle on depression (forced swim test), anxiety (forced swim test and horizontal bar tests), and motor coordination (horizontal bar test and rotarod). (**A**,**B**) Immobility time (**A**) and number of immobile episodes (**B**) in the forced swim test. (**C**) Latency to fall on the rotarod for two days of training is plotted. The numbers (1, 2) represent days of testing. (**D**–**L**) The horizontal bar tests. (**D**,**G**,**J**) Time to the safety box, (**E**,**H**,**K**) and number of pushes required, and (**F**,**I**,**L**) the number of foot slips on the first two days of training and 24 h after training on 4 cm, 2 cm, and 1 cm bars. All, *n* = 13–16, ANOVA or mixed measures ANOVA. * *p* < 0.05; *p*-values indicated numerically when *p* < 0.1.

DFP-treated animals (both VEH and SAR) spent less time immobile and had fewer immobile episodes compared to the control groups, though this was not significant (Figure 8A,B). On the rotarod, a measure of motor coordination, there were no differences between days one and two of training or between groups for the latency to fall (Figure 8C). On the horizontal bar test, we did not see any differences in the number of foot slips on any of the bars between the groups (Figure 8F,I,L). We did, however, see differences in how fast animals crossed the beam, which may indicate anxiety or impulsivity indirectly (see Discussion). All groups reached the goal-box significantly faster on the second day of training (4 cm beam) and required fewer pushes compared to the first day of training (Figure 8D,E). Interestingly, the PBS and VEH, DFP and VEH, and DFP and SAR all had fewer footslips on the second day compared to the first day (Figure 8F). After training, animals were then tested the next day using a 2 cm bar. Interestingly, DFP and VEH animals reached the goal significantly faster than PBS and SAR animals (Figure 8G), and there were no differences in the number of foot slips between groups in both 2 cm and 1 cm bars (Figure 8I,L). DFP and SAR animals took more time to reach the goal, but this was not significant. The same trends, as with the 2 cm bar, were observed for the number of pushes animals required to walk across the beam (Figure 8G). When animals were tested on the 1 cm beam, there were no significant differences between the groups for both the time it took animals to reach the goal and the number of pushes that were required (Figure 8H,I).

### 3.5. Long Term Impact of DFP and Mitigation by SAR on Gliosis and Neurodegeneration in Animals with ~20 min of Continuous CS during SE

Following behavioral tests, animals were given 3–4 days of rest (about 10 weeks after DFP intoxication) and then were euthanized to assess gliosis and neurodegeneration, as in the previous cohort of mixed-sex. One-half of the animals were perfuse-fixed with 4% PFA for IHC, while the other half of the animals were used for Western blotting without perfusion.

#### 3.5.1. Microgliosis

**Figure 9:**
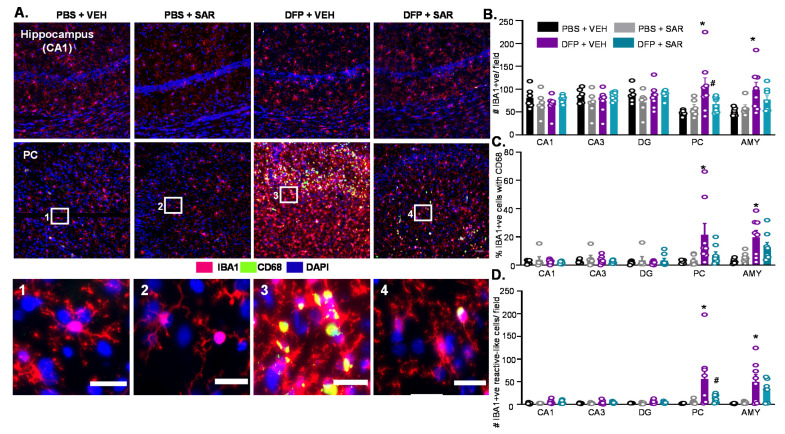
Microgliosis 10 weeks post-DFP (~20 min of SE) and mitigation by SAR. (**A**) Representative IHC images of IBA1 and CD68 positive cells from each treatment group. Scale, 50 μm for whole field (0.44 m^2^) images and 25 μm for boxed images (1–4). (**B**–**D**) Cell quantification. (**B**) Number of IBA1 positive cells. (**C**) Percent IBA1 positive cells colocalized with CD68 positive cells. (**D**) Number of IBA1 positive cells with reactive-type morphology. Reactive cells were considered to have large cell bodies and retracted processes. All, *n* = 6–8, mixed measures ANOVA. * *p* < 0.05 compared to at least one control group # *p* < 0.05 compared to DFP + VEH (B-G).

Representative IHC images of IBA1 and CD68 staining are shown in Figure 9A. Interestingly, in the hippocampus of the DFP and VEH group, there were no differences between the groups for the total number of IBA1 positive cells (Figure 9B), percent of IBA1 positive cells with CD68 (Figure 9C), or the number of IBA1 positive cells with reactive morphology (Figure 9D). However, we found a significant increase in all of these parameters for DFP- and VEH-treated animals in the PC and the AMY (Figure 9B–D). SAR treatment led to a reduction of IBA1 positive cells compared to DFP- and VEH-treated animals in the PC (significant) and AMY (not significant) (Figure 9B). Similarly, there was also a reduction in IBA1 cells with reactive morphology in DFP and SAR animals compared to DFP and VEH animals in the PC (significant) and AMY (not significant) (Figure 9D). DFP- and SAR-treated animals also had a reduction in the percent of CD68 positive cells compared to DFP and VEH in the PC and AMY, but the reduction was not statistically significant (Figure 9C).

#### 3.5.2. Astrogliosis

**Figure 10:**
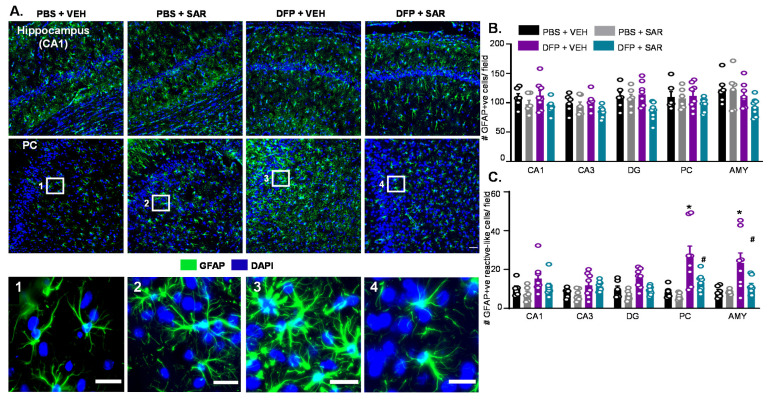
Astrogliosis 10 weeks post-DFP (~20 min of SE) and mitigation by SAR. (**A**) Representative IHC images of GFAP positive cells from each treatment group. Scale, 50 μm for whole field (0.44 m^2^) images and 25 μm for boxed images (1–4). (**B**,**C**) Cell quantification. (**B**) Number of GFAP positive cells. (**C**) Number of GFAP positive cells with reactive-type morphology. Reactive cells were considered to have large cell bodies and retracted processes. All, *n* = 6–8, mixed measures ANOVA. * *p* < 0.05 compared to at least one control group # *p* < 0.05 compared to DFP + VEH (B-G).

Representative images from the hippocampus and AMY for GFAP are shown in Figure 10A. There was no difference in the number of GFAP positive cells between groups (Figure 10B). There was only a significant difference in the number of GFAP positive cells with reactive morphology in the AMY and PC but not in the hippocampus (Figure 10C). There was significant mitigation by SAR of DFP-induced change in GFAP morphology in both the AMY and PC (Figure 10C).

#### 3.5.3. Neurodegeneration

**Figure 11:**
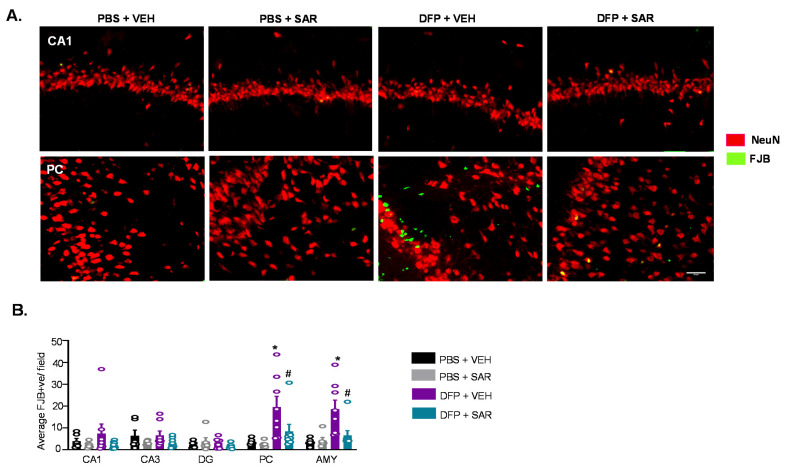
Neurodegeneration 10 weeks post-DFP (~20 min of SE) and mitigation by SAR. (**A**) Representative IHC images for NeuN and FJB positive cells from each treatment group. Scale, 50 μm. (**B**) Number of FJB positive cells (*n* = 6–8, mixed measures ANOVA * *p* < 0.05 or *p*-values indicated numerically when *p* < 0.1 compared to at least one control group, # *p* < 0.05 compared to DFP + VEH (B-G). Field is 0.44 m^2^.

We further analyzed the number of FJB positive cells; representative images are shown in Figure 11A. In DFP and VEH animals, there was a significant upregulation of FJB positive cells in the PC and AMY, but not in the hippocampus, compared to controls (Figure 11B). This was significantly mitigated by SAR treatment.

### 3.6. SAR Mitigates DFP-Induced Toxicity as an Antioxidant

Considering the significant mitigation of neurodegeneration and reactive gliosis by SAR, we hypothesized that SAR could target nitroxidative stressors to achieve its neuroprotective role as an antioxidant. We utilized IHC and Western blotting to determine the expression levels of inducible nitric oxide synthase (iNOS), 3-nitrotyrosine (3NT) and GP91^phox^ (a subunit of NADPH oxidase). We hypothesized that the number of positive iNOS, 3NT, and GP91^phox^ cells might be associated with glial activation. Therefore, we used a simple linear regression to determine the correlation between the markers of oxidative stress and the number of reactive microglia and astrocytes in each treatment group. These regressions are outlined in Tables S2–S4.

#### 3.6.1. SAR Mitigates DFP-Induced iNOS

**Figure 12:**
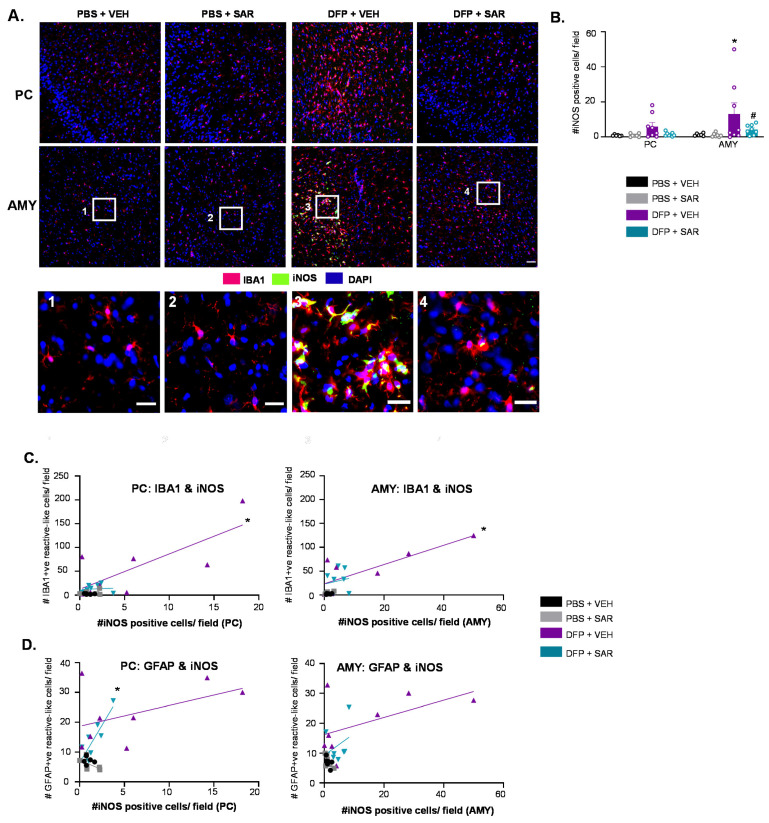
iNOS expression 10 weeks post-DFP (~20 min of SE) and mitigation by SAR. (**A**) Representative IHC images of iNOS and IBA1 positive cells from each treatment group. Scale, 50 μm for whole field (0.44 m^2^) images and 25 μm for boxed images (1–4). (**B**) Number of iNOS positive cells (*n* = 6–8, mixed measures ANOVA). (**C**) Number (#) of iNOS positive cells compared to the number of IBA1 positive cells with reactive type morphology (*n* = 6–8, simple linear regression). (**D**) Number of iNOS positive cells compared to the number of GFAP positive cells with reactive type morphology (*n* = 6–8, simple linear regression). * *p* < 0.05 compared to at least one control group or a positive correlation, # *p* < 0.05 compared to DFP + VEH (B-G).

Representative images of iNOS for each treatment group are shown in Figure 12A. iNOS was primarily localized in microglia (IBA1 positive cells); we therefore only counted iNOS positive cells that were colocalized with IBA1. There was a significant upregulation of iNOS in DFP- and VEH-treated animals in the AMY with significant mitigation by SAR (Figure 12B). There was also upregulation of iNOS in the PC, but this was not significant. We hypothesized that the degree of iNOS positive cells might be indicative of the degree of glial activation. A simple linear regression showed a significant correlation between the number of IBA1 positive cells with reactive morphology and the number of iNOS positive cells in the DFP and VEH group, but not the other groups (Figure 12C, PC, R^2^ = 0.577, AMY R^2^ = 0.660). There was also a significant correlation between the number of iNOS positive cells and GFAP positive cells with reactive morphology in the PC of the DFP and SAR group (Figure 12D, R^2^ = 0.737).

#### 3.6.2. SAR Mitigates DFP-Induced 3NT

**Figure 13:**
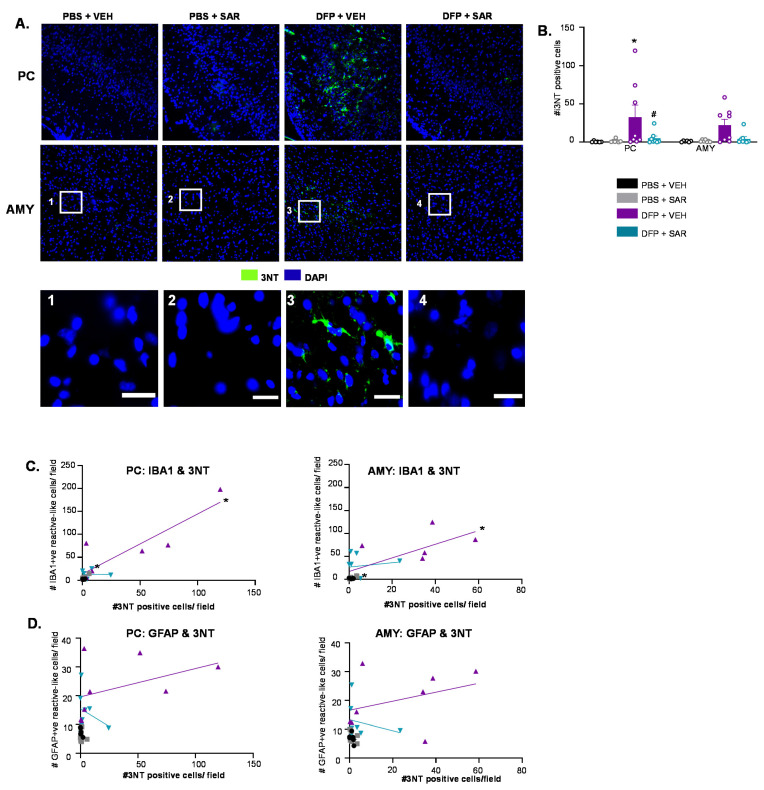
3NT expression 10 weeks post-DFP (~20 min of SE) and mitigation by SAR. (**A**) Representative IHC images of 3NT positive cells from each treatment group. Scale, 50 μm for whole field (0.44 m^2^) images and 25 μm for boxed images (1–4). (**B**) Number (#) of 3NT positive cells (*n* = 6–8, mixed measures ANOVA). (**C**) Number of 3NT positive cells compared to the number of IBA1 positive cells with reactive type morphology (*n* = 6–8, simple linear regression). (**D**) Number of 3NT positive cells compared to the number of GFAP positive cells with reactive type morphology (*n* = 6–8, simple linear regression). * *p* < 0.05 compared to PBS + VEH and # *p* < 0.05 compared to DFP + VEH (**B**), * *p* < 0.05 significant correlation (**C**,**D**).

Representative IHC images of 3NT for each treatment group are shown in Figure 13A. There was little-to-no staining in control animals and DFP and SAR animals. There was upregulation of 3NT in the DFP and VEH in both the PC and AMY (Figure 13B). In the PC, SAR significantly mitigated DFP-induced upregulation of 3NT (Figure 13B). Following a simple linear regression analysis, we found a significant correlation between the number of 3NT positive cells and the number of reactive type IBA1 positive cells for the DFP and VEH group in both the PC (R^2^ = 0.784) and AMY (R^2^ = 0.541) (Figure 13C). This was similar for the DFP and SAR group in the PC (R^2^ = 0.856) and AMY (R^2^ = 0.825) (Figure 13D). Interestingly, there was no significant correlation for any group in any location between the number of 3NT positive cells and reactive type GFAP positive cells. There were no significant changes in the expression of 3NT in the PC/AMY region, as revealed by Western blotting (Figure S2A,B).

#### 3.6.3. Impact of DFP and SAR on GP91^phox^

**Figure 14:**
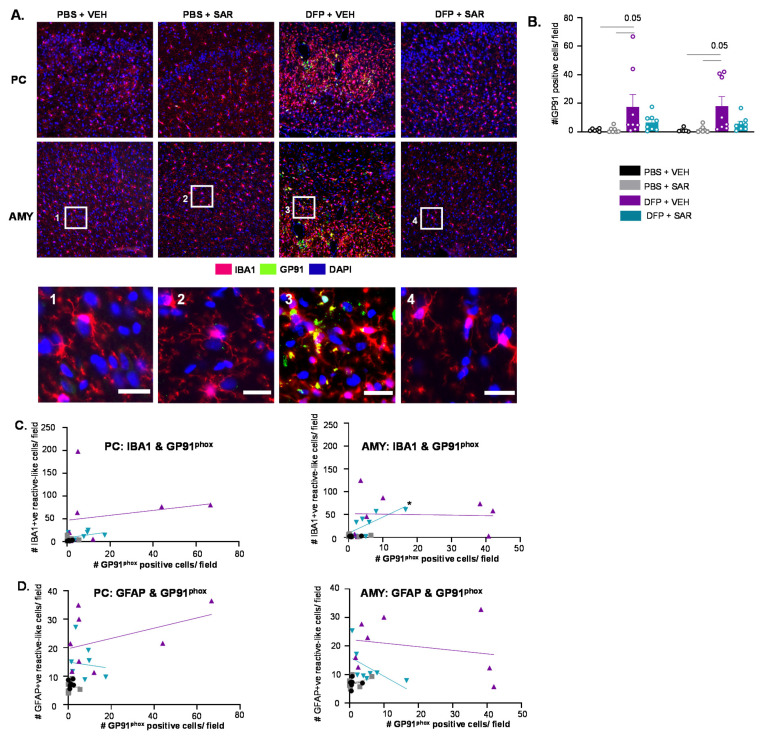
GP91^phox^ expression 10 weeks post-DFP (~20 min of SE) and mitigation by SAR. (**A**) Representative IHC images of GP91^phox^ positive cells from each treatment group. Scale, 50 μm for whole field (0.44 m^2^) images and 25 μm for boxed images (1–4). (**B**) Number (#) of GP91^phox^ positive cells (*n* = 6–8, mixed measures ANOVA). (**C**) Number of GP91^phox^ positive cells compared to the number of IBA1 positive cells with reactive type morphology (*n* = 6–8, simple linear regression). (**D**) Number of GP91^phox^ positive cells compared to the number of GFAP positive cells with reactive type morphology (*n* = 6–8, simple linear regression; *p*-values indicated numerically when *p* < 0.1).

Representative IHC images of GP91^phox^ are shown in Figure 14A. Many of the GP91^phox^ cells colocalized with IBA1 positive cells in the PC and AMY; we, therefore, counted GP91^phox^ positive cells that were colocalized with IBA1. There was a non-significant upregulation of GP91^phox^ positive cells in DFP and VEH animals compared to controls with mitigation by SAR, though the difference was not significant (Figure 14B). Following simple linear regression, we found a correlation between GP91^phox^ positive cells and IBA1 positive cells with reactive type morphology in the AMY of DFP- and SAR-treated animals but not any other group or location (Figure 14C). There were no significant correlations between the number of GP91^phox^ positive cells and the number of GFAP positive cells with reactive type morphology (Figure 14D). There was no difference in GP91^phox^ in the PC/AMY region between the treatment groups as revealed by Western blotting (Figure S2C,D).

## 4. Discussion

The purpose of this study was to determine the disease-modifying potential of SAR in an OP model of epilepsy in both males and females. SAR is a potent inhibitor of SFKs, which include Src, Yes, Trk, Fgr, Hck, Blk, Lck, Lyn, and Fyn. SAR has an IC50 of 2.7 nM and 10 nM against c-Src and Fyn, respectively, when tested in vitro [83]. SAR and other SFK inhibitors were first used in cancer models and have been found to be well tolerated in patients with solid tumors; 175 mg once daily was found to be the maximum tolerable dose [84,85]. Notably, we used 20 mg/kg once daily, which is equivalent to about 195 mg for the average-weight human. Human studies have shown some adverse effects to SAR administration, such as fatigue, nausea, diarrhea, and headaches [67,86]. Notably, we did not observe any side effects in this study with 20 mg/kg daily dose, unlike our previous study (using 25 mg/kg twice a day for the first three days) in the DFP model, where SAR-treated animals had increased weight loss and mortality compared to VEH-treated animals [70]. Although SAR treatment did not significantly mitigate weight loss or morbidity measures (Irwin analysis), it appears that 20 mg/kg is more tolerable in these animals. The mortality in the SAR group could be due to off-target effects from the early doses of SAR’s reactive metabolites as discussed in our previous publication [70]. For example, some studies in the rat and human livers detected oxidative metabolites after exposure to SAR [87,88]. SAR is also bio-activated by P450 3A4 [87]. Interestingly, P430 3A4 is also required for the metabolism of MDZ, which was administered two hours prior to MDZ [89]. Since we have been dosing SAR orally at 20 mg/kg as a single dose/day, it is likely that rapidly accumulated SAR metabolites in the liver from the first 2–3 doses combined with DFP-induced gastrointestinal changes may have contributed to mortality in some animals [90]. Diet incorporation of SAR may achieve therapeutic concentrations of SAR gradually and could mitigate/minimize toxicity that normally occurs due to the rapid rise of SAR’s metabolites in the liver. Alternatively, starting with a low dose in the first few days post-DFP followed by a dose-ramping approach could be beneficial.

SFK inhibition has been tested in a variety of animal models of neurological diseases. Importantly, our previous work and others’ have demonstrated SAR’s ability to cross the BBB in rodents [57,91]. SAR is also an inhibitor of the ATP-binding cassette transporter, ABCG2, which facilitates SAR to cross the BBB compared to the other SFK inhibitors [92,93]. For example, one study administered SAR in mice with the genetic background of AD and found that SAR mitigated deficits on the MWM when SAR treatment was prolonged for 3–5 weeks in contrast to a shorter treatment course (1–2 weeks) [91]. Similar results were shown in both the MWM and novel object test even when treatment was stopped eight days prior to testing [94]. A recent study used a new PET tracer (^11^C-UCB-J-PET) in wild-type and AD mice and found that SAR treatment mitigated the loss in synaptic density [95]. There is also evidence for the beneficial effects of SFK inhibition in PD. In α-synuclein PD model, SAR administration eight weeks after disease initiation attenuated the propagation of the α-synuclein fibrils [96].

SAR has been tested in various clinical trials, including for breast cancer [86], prostate cancer [97], osteosarcoma, [98] and several others. Following its testing in cancer, SAR has also been tried in several models (both animal and human) of neurological diseases. One study administered 50–125 mg daily for four weeks to patients with probable AD and assessed tolerability as well as cognitive measures, via previously established scales [67]. There was no significant effect by SAR on any measure. Another study, using patients with mild AD dementia, administered 100–125 mg daily for a year [99]. Again, no significant improvement was observed for any clinical measure. The discrepancy between the success in animal models versus the lack of efficacy in clinical trials may be attributed to the level of disease progression and severity or length of SAR treatment. As many of the disease progression mechanisms in AD and PD are shared with those in epilepsy, we began to investigate SFK inhibition by SAR in epilepsy models before the onset of epilepsy as a preventative approach [70,72].

We showed that SAR treatment (25 mg/kg twice a day for three days followed by once a day for four days) shortly after the initiation of SE by KA in rats reduced the occurrence of SRS, spike frequency, as well as proinflammatory cytokines in the hippocampus and serum [68]. Another study also found reduced SRS and neuronal loss in SAR-treated animals following SE induced by pilocarpine [69]. This led to the hypothesis that SAR might be effective in preventing epileptogenesis and neurodegeneration in DFP-induced SE models too. We recently tested SAR in animals that had ~20 min CS during SE and found that at eight days post-challenge, there was a reduction in SRS while handling during the treatment period, as well as gliosis and neurodegeneration [70]. However, in the previous study, we found increased morbidity in SAR-treated animals that had severe SE compared to the VEH-treated animals due to the repeated dosing regimens. Therefore, we decided to reduce the dose of SAR in the current study and tested SAR in both 1 h and ~30 min SE models to determine the therapeutic potential of the drug.

Our previous studies showed a reduction of SRS in SAR-treated animals, which were recorded while handling during the treatment period [68,69]. Seizures can occur during handling due to the stress of injections, oral gavage, and experimenter fear. Stress increases cortisol levels, which promotes epileptogenesis [100,101,102]. Though the handling method does provide a snapshot of seizure activity, continuous video EEG would be ideal for a robust assessment as we did in our previous studies [103,104]. In this study, we found a reduction in the number of animals that had at least one CS regardless of SE duration. Notably, in animals with ~60 min CS, none of the SAR-treated males and 17% of the SAR-treated females had at least one CS. This could be due to a sex difference in the SAR efficacy but could also be due to the increased dose of DFP (5 mg/kg vs. 4 mg/kg), though notably, there was no difference in SE severity between sexes. The rationale for increasing the dose of DFP in females in this study was due to the decreased SE severity to the DFP (that we had at that time in our previous studies [26]. Whether the variability in response to DFP in females was due to the source of DFP or vendors or housing (females were housed in a separate room than males in this study) needs further investigation.

Though seizure activity is the primary measurement of interest when studying epilepsy, gliosis and neurodegeneration are also important disease hallmarks [42,48,105,106,107,108]. DFP, like other OPNAs, leads to the development of SE through the irreversible inhibition of AchE [109]. SE, regardless of etiology, leads to gliosis and neurodegeneration, which in turn contributes to epileptogenesis [110,111,112]. In this study, when the animals were given MDZ one hour after DFP and sacrificed at eight days, there was significant upregulation of microgliosis, astrogliosis, and neurodegeneration in all regions of the brain as expected in the DFP model [26,28,30,31,70,113]. However, in this group of rats, there was a limited impact of SAR on gliosis and neurodegeneration. We found a significant reduction in the number of IBA1 positive cells in the female AMY and a reduction in reactive astrogliosis in males (in the hippocampus). This finding indicates that there may be a sex difference in the efficacy of SAR in mitigating DFP-induced reactive astrogliosis. This could also be the consequence of the increased dose used in females, although they did have equal initial SE severity compared to males. Notably, astrocytes contain a large number of receptors for gonadal hormones, including those for estrogen (ERα and Erβ), which controls the intracellular calcium levels [114,115]. Perhaps hormonal differences between males and females play a role in determining the efficacy of SAR, which requires further studies. Furthermore, it is unclear whether a prolonged washout time is required to determine the real effects of SAR.

Having observed a limited effect of SAR treatment (for a week) in the 1 h SE model, we decided to reduce the duration of SE to understand whether the SE severity impacts SAR efficacy while maintaining the treatment duration for a week (~20 min group). Interestingly, in this cohort, DFP only led to a significant increase in gliosis and neurodegeneration in the PC and AMY region, not in the hippocampus, which was similar to the findings at day eight post-DFP in our recent study [70]. Traditionally, the hippocampus has been considered the focal point of epileptogenesis in animal models and humans [116,117,118]. Due to the interconnected nature of the brain regions studied, it would be reasonable to assume that the neuronal and glial changes may spread to other brain regions over time as the disease progresses [119,120]. However, our study suggests that in the ~20 min of SE in the DFP model, the damage is limited to the PC/AMY region and not the hippocampus.

SAR treatment for a week significantly reduced gliosis and neurodegeneration in the PC and AMY compared to the VEH-treated animals in 10 weeks post-DFP group. The two cohorts of animals used in this study (8 days post-DFP and 10 weeks post-DFP) were only different in initial SE severity and the time point of sacrifice post-DFP. It is possible that SAR treatment had an overall “washout” effect in 10 weeks post-DFP group and mitigated pathology. More likely, however, the overall reduction in SE duration improved the efficacy of SAR in mitigating gliosis and neurodegeneration. Our previous study and the studies from others have shown that the degree of neuropathology depends on the initial SE severity and duration [30,38,70], suggesting that SAR’s efficacy also depends on the initial SE severity in epilepsy. As hippocampal sclerosis is common in human patients with temporal lobe epilepsy, it would be useful to further optimize SAR efficacy by extending the duration of its dosing regimen in animals with longer SE to target the hippocampus [121,122].

Prior to euthanasia, we assessed the second cohort of animals (~20 min CS) for behavioral comorbidities, as epilepsy is associated with changes in learning, memory, anxiety, depression, and motor coordination [123,124,125,126]. Importantly, none of these tests, nor brain pathology, showed significant changes in animals treated with SAR on its own (without DFP challenge). This suggests that there were no obvious side effects of SAR treatment per se with the dosing regimen tested in this study. We used the novel object test as well as the MWM to assess learning and memory. Overall, most of these tests did not reveal significant effects nor significant mitigation of DFP-induced toxicity by SAR, but we still find it useful to discuss the trends in the context of other studies.

The novel object test primarily involves the hippocampus for object recognition and memory consolidation [127,128]. In the novel object recognition test, we saw a non-significant (*p* = 0.09) reduction in the discrimination index in DFP-treated animals. Since the hippocampus was largely unaffected in the ~20 min SE group, it is likely that other brain regions may have a role in object recognition. For example, one study found that infusing norepinephrine into the basolateral AMY led to enhanced long-term memory retention and that blocking with propranolol impaired object recognition [129]. Perhaps SE-induced injury to the AMY by DFP may have affected performance in the novel object recognition test. There was mitigation (not significant) by SAR when animals were tested 3 h after familiarization in the novel object test. However, modification of the NOR test may yield better outcomes, which are currently under investigation. Further tests are needed to make conclusions about its disease modifying effect.

In the MWM, there was no difference in the learning curve between groups, which contradicts previous studies from other groups showing reduced learning in animals exposed to OPs or other chemoconvulsants [130,131,132]. Since ~20 min was not enough to cause significant hippocampal damage in the rat DFP model, and MWM is also a hippocampal-dependent task, we did not observe any differences in the test results [133,134,135]. Notably, DFP- and SAP-treated animals did spend significantly more time in the target quadrant compared to the opposite quadrant, which suggests some interaction between DFP and SAR and memory consolidation. This needs to be explored further.

We utilized the forced swim test to assess anxiety and depression in these animals [77]. Interestingly, there were no differences between the groups in the forced swim test, which conflicts with previous studies that the animals exposed to chemocovulsants were more immobile [136]. Notably, we used an apparatus with a larger diameter (28 cm versus 20 cm) than previously reported, which may have allowed the animals to swim in a wider area. The apparatus in our study was also opaque, and animals were tracked from above rather than from a transparent cylinder from the side [137]. This may have led to differences in the sensitivity of tracking immobility. Future experiments may be needed with a smaller diameter apparatus with an additional camera from the side.

The horizontal bar test is typically used to assess motor ability by measuring the number of foot slips or the time taken to reach the target while an animal is walking across the beam [138]. We did not find an effect of treatment on the number of foot slips, which indicates that DFP-induced SE does not lead to changes in motor ability. This is confirmed by the lack of difference between groups in the rotarod, which is a well-accepted measure of motor ability in rodents [139]. By observation, it appeared that many of the rats fell off the rod due to distraction rather than a lack of ability to stay on the beam. Future tests might extend the training period or change the initial speed to mitigate these effects. Furthermore, of note, in the horizontal bar test, all treatment groups improved their time to reach the safety box on the first day compared to the second day, which suggests there was no effect of treatment on motor learning. There is some evidence that epilepsy leads to a change in motor ability, though mostly in children [126,140]. Possibly, there is no change in gross motor behavior in these animals, or the deficits are more present in fine motor skills.

In the horizontal bar test, we did observe that on the 2 cm beam, DFP-treated animals reached the platform significantly faster. We interpret this as an increased fear of the height (anxiety), driving the animals to reach the platform more quickly or increased impulsivity. Once trained, control animals were more likely to stop and explore their surroundings on the beam before moving to the platform. This is exhibited in both the time to reach the platform as well as the number of pushes by an experimenter. A mouse study showed that the neurons in the basolateral AMY responds to height [141]. Although we did not quantify the subregions of AMY, it is likely that the loss of neurons in the AMY in our model extinguished the fear and anxiety in these animals. The AMY consists of a number of interconnected nuclei [142]. There was no difference in the time to the safety box when animals were later tested on the 1 cm beam, likely because the bar was too small for even controls of this age to walk across without many experimenters pushing. Overall, assessment of behavioral comorbidities showed some deficits in the animals exposed to DFP and minimal mitigation by SAR.

Overall, in animals with ~20 min CS, SAR mitigated CS during handling, neuroinflammation, neurodegeneration, and non-significantly reduced some behavioral comorbidities. Many studies have investigated the glial and neuronal mechanisms of SFKs in disease onset and progression, and we have recently reviewed the previous work and discussed the mitigation strategies by SFK inhibitors [48]. In neurons, Fyn kinase can modulate glutamatergic receptors, including NMDARs (NR2A and NR2B subunits), as well as metabotropic glutamatergic receptors, which contribute to hyperexcitability [49,52,55,143]. Fyn is known to phosphorylate tau, which facilitates the migration of the Fyn–Tau complex to the synaptic site and phosphorylates the glutamatergic receptors [53]. In addition to glutamatergic signaling, Fyn can also modulate GABAergic receptors function, which plays a role in the regulation of seizure activity [144].

In glial cells, Fyn and Src are implicated in neuroinflammation pathways following neurological insult. For example, phosphorylated Fyn and Src interact with the Pyk2/paxillin complex, which is essential for microglial migration [145,146]. Therefore, inhibition of SFKs could prevent microglia from becoming reactive, following injury, to protect vulnerable neurons. In a PD model, it was shown that phosphorylated Fyn could activate PKCδ, which leads to translocation of NFκB to the nucleus [58,147]. NFκB mediates the transcription of several genes, including proinflammatory cytokines and mediators of oxidative stress such as iNOS and NADPH oxidase (NOX) [59,61,148,149]. These findings led to the hypothesis that anti-oxidant pathways play a role in SAR-mediated mitigation of DFP-induced toxicity. Therefore, we utilized IHC and Western blotting to determine the impact of DFP and SAR on the markers of oxidative stress. As we only detected changes in gliosis and neurodegeneration in the PC/AMY region, we focused our analysis of oxidative stress on these regions.

iNOS is one of three isoforms of NOS, which converts L-arginine to nitric oxide (NO) [150]. iNOS, in contrast to other isoforms (nNOS and eNOS), is not constitutively active and produces high concentrations of NO in short pulses in response to injury [151,152]. We have previously shown the upregulation of iNOS following DFP intoxication and mitigation by an iNOS inhibitor, 1400 W [28]. Notably, Src phosphorylates iNOS and stabilizes its half-life, which might contribute to iNOS-mediated toxicity [153]. Future studies are required to understand the post-translational modifications of iNOS and mitigation by SAR. In this study, we showed the upregulation of iNOS in both the PC and AMY and, usually, iNOS positive cells were found in clusters within these regions. There was also an increase in 3-NT in both the PC and AMY though it was only significant in the PC, opposite to what we found with iNOS. 3-NT is a post-translational modification in which a NO_2_ group is added to various proteins [154]. NO, which can be generated by NOS, can be further oxidized to NO_2_, meaning that the presence of iNOS should also lead to increased 3NT levels. Possibly the NO produced by iNOS-positive cells elsewhere, for example, the neighboring AMY, may contribute to higher levels of 3-NT in PC. Alternatively, NO production mediated by nNOS and eNOS may also contribute to the increased 3-NT levels. GP91^phox^ is the catalytic subunit of NADPH oxidase (NOX). NOX2 is predominantly expressed in the brain [155]. Following neurological insult, other cytosolic domains form a complex with membrane-bound GP91^phox^, leading to the transfer of an electron to oxygen to form the free radical peroxide (O_2_^−^) [156,157]. In this study, we found a non-significant increase in GP91^phox^ in both the AMY and PC, which aligns with previous studies. Similar to iNOS, 3NT and GP91^phox^ were also typically found in clusters within the PC/AMY. This might explain why there were no significant differences in 3-NT or GP91^phox^ following Western blotting. The entire PC/AMY region was dissected, which likely diluted the effects of clustered upregulation of these markers. There was mitigation by SAR in most of these regions for the presence of these markers, which suggests that SFKs play a role in the upregulation of oxidants following exposure to DFP. We measured these markers ten weeks after DFP intoxication (9 weeks post-cessation of SAR administration), which implies that there was SAR-induced mitigation of DFP-induced oxidative stress that persisted well past the point of administration.

All three of the markers that we tested (iNOS, 3-NT and GP91^phox^) are considered to be valid markers of oxidative stress and the presence of free radicals. Free radicals such as NO and O_2_^−^ are essential for many cellular processes, including homeostasis, cellular growth, metabolism, and much more [158,159]. In the context of neurological disease, however, these regulators can become deleterious [160,161]. For example, NO and O_2_^−^ can form peroxynitrite (ONOO^−^), which can nitrosylate proteins and lead to the misregulation of important signaling pathways [152]. Free radicals contribute to neuroinflammation, which in turn exacerbates seizures [162]. We, therefore, hypothesized that these markers might be correlated with the degree of glial activation. In many regions, there was a correlation between these markers of oxidative stress and glial morphology, which supports our hypothesis, especially in the DFP and VEH groups.

## 5. Conclusions

In summary, the goal of this study was to determine the disease-modifying effects of SAR, an SFK inhibitor, in mitigating the neurotoxic effects of DFP-induced SE. We found that the severity and duration of SE and the duration of SAR treatment are important factors to determine the therapeutic potential of the drug. Two cohorts of animals were used in this study. In one group, animals had about an hour SE; in this group, there was a minimal impact of SAR on DFP-induced toxicity due to prolonged duration of CS and a week-long SAR treatment with only 24 h washout time. Future studies with long-term treatment may increase the efficacy of SAR. Alternatively, SAR could be introduced into the diet instead of direct oral dosing. In animals with about 20 min SE, SAR mitigated DFP-induced changes in gliosis and neurodegeneration. SAR appears to induce neuroprotective effects, at least in part, via an antioxidant mechanism.

## Figures and Tables

**Table 1:** Number of animals by sex and sacrifice time-point. Number in parenthesis indicates mortality.

Timepoint	Midazolam Administration	PBS + VEH	PBS + SAR	DFP + VEH	DFP + SAR
8 days Males	One hour post DFP	5	0	7	10 (20%)
8 days Females	One hour post DFP	5	0	8	8 (25%)
10 weeks Males	After 20 min CS	14	14	16	16 (6%)

## Data Availability

The data will be made available by the authors following an email to the corresponding author: tswamy@iastate.edu. The data are not publicly available due to a pending PhD thesis.

## Supplementary Materials

The following are available online at https://www.mdpi.com/article/10.3390/antiox11010061/s1, Figure S1: No evidence of pre-existing preference for objects used in the novel object recognition test. Figure S2: Western blotting 3-NT and GP91phox. Table S1: List of antibody and antibody concentrations. Tables S2–S4: Summary of correlational data.
