# Supplementary material for: Saracatinib, a Src Tyrosine Kinase Inhibitor, as a Disease Modifier in the Rat DFP Model: Sex Differences, Neurobehavior, Gliosis, Neurodegeneration, and Nitro-Oxidative Stress
Source: Antioxidants (Basel). 2021 Dec 28;11(1):61. doi: 10.3390/antiox11010061 (PMC8773289; doi:10.3390/antiox11010061)
Supplement: Supplementary file 1 [file antioxidants-11-00061-s001.zip › Supplementary material/Supplemental figures and tables.pdf]

Figure S1

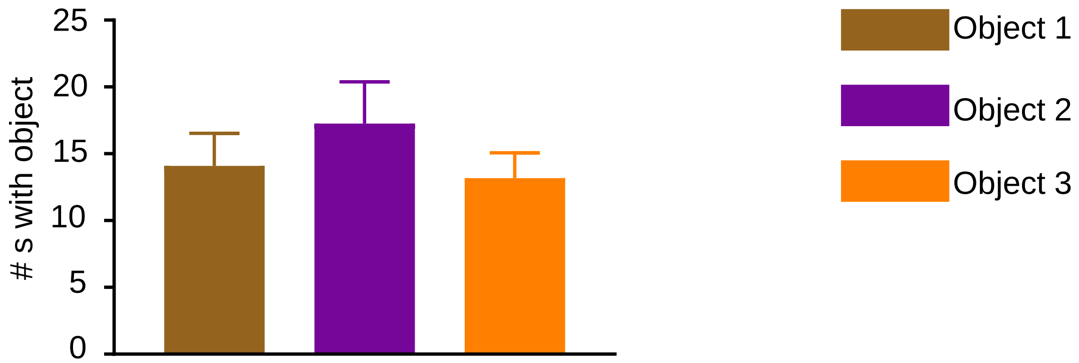

Figure S2

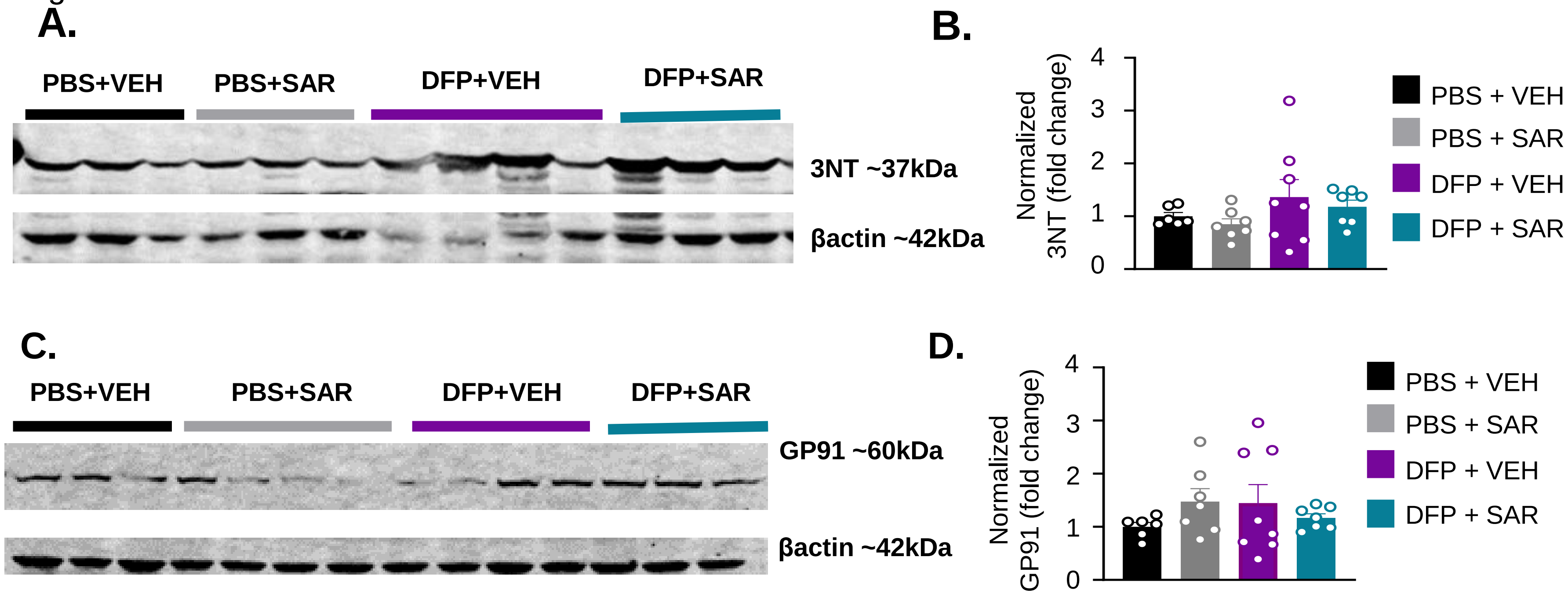
A.
PBS+VEH
PBS+SAR
DFP+VEH
DFP+SAR
3NT ~37kDa
βactin ~42kDa
B.
Normalized
3NT (fold change)
0
1
2
3
4
PBS + VEH
PBS + SAR
DFP + VEH
DFP + SAR
C.
PBS+VEH
PBS+SAR
DFP+VEH
DFP+SAR
GP91 ~60kDa
βactin ~42kDa
D.
Normalized
GP91 (fold change)
0
1
2
3
4
PBS + VEH
PBS + SAR
DFP + VEH
DFP + SAR

Table S1. Antibody suppliers and concentrations

Antibody	Species	Supplier	Concentration	
			IHC	WB
IBA1	Anti-goat	Abcam	1:400	
GFAP	Anti-mouse	Sigma Aldrich	1:400	
CD68	Anti-rabbit	Abcam	1:300	
NeuN	Anti-rabbit	Millipore	1:400	
iNOS	Anti-rabbit	Abcam	1:100	
3-NT	Anti-mouse	Abcam	1:100	1:1000
β-actin	Anti-rabbit	Sigma Aldrich		1:10000
β-actin	Anti-mouse	Sigma Aldrich		1:10000
$GP91^{phox}$	Anti-mouse	Santa Cruz	1:100	1:1000
Biotinylated	Anti-goat	Jackson Immunoresearch	1:300	
Biotinylated	Anti-rabbit	Jackson Immunoresearch	1:300	
Alexaflour	Anti-mouse	Jackson Immunoresearch	1:80	
Alexaflour	Anti-rabbit	Jackson Immunoresearch	1:80	
Streptavidin CY3		Jackson Immunoresearch	1:300	
IRDye 800CW	Anti-rabbit	LI-COR Biosciences		1:10000
IRDye 800CW	Anti-mouse	LI-COR Biosciences		1:10000
IRDye 800CW	Anti-goat	LI-COR Biosciences		1:10000
IRDye 680LT	Anti-mouse	LI-COR Biosciences		1:10000
IRDye 680LT	Anti-rabbit	LI-COR Biosciences		1:10000

Table S2. Summary of Regression for Glial cell markers and iNOS. *Indicates p<0.05

Glial cell marker	Oxidative stress marker	Region	Experimental Group	R^2
IBA1	iNOS	PC	PBS+VEH	0.073
IBA1	iNOS	PC	PBS+SAR	0.163
IBA1	iNOS	PC	DFP+VEH	**0.577***
IBA1	iNOS	PC	DFP+SAR	0.007
IBA1	iNOS	AMY	PBS+VEH	0.109
IBA1	iNOS	AMY	PBS+SAR	0.479
IBA1	iNOS	AMY	DFP+VEH	**0.660***
IBA1	iNOS	AMY	DFP+SAR	0.028
GFAP	iNOS	PC	PBS+VEH	0.033
GFAP	iNOS	PC	PBS+SAR	0.33
GFAP	iNOS	PC	DFP+VEH	0.225
GFAP	iNOS	PC	DFP+SAR	**0.737***
GFAP	iNOS	AMY	PBS+VEH	0.336
GFAP	iNOS	AMY	PBS+SAR	0.45
GFAP	iNOS	AMY	DFP+VEH	0.28
GFAP	iNOS	AMY	DFP+SAR	0.117

Table S3. Summary of Regression for Glial cell markers and 3NT. *Indicates p<0.05

Glial cell marker	Oxidative stress marker	Region	Experimental Group	R^2
IBA1	3NT	PC	PBS+VEH	0.037
IBA1	3NT	PC	PBS+SAR	**0.856***
IBA1	3NT	PC	DFP+VEH	**0.784***
IBA1	3NT	PC	DFP+SAR	0.000
IBA1	3NT	AMY	PBS+VEH	0.260
IBA1	3NT	AMY	PBS+SAR	**0.825***
IBA1	3NT	AMY	DFP+VEH	**0.541***
IBA1	3NT	AMY	DFP+SAR	0.024
GFAP	3NT	PC	PBS+VEH	0.587
GFAP	3NT	PC	PBS+SAR	0.018
GFAP	3NT	PC	DFP+VEH	0.194
GFAP	3NT	PC	DFP+SAR	0.010
GFAP	3NT	AMY	PBS+VEH	0.218
GFAP	3NT	AMY	PBS+SAR	0.163
GFAP	3NT	AMY	DFP+VEH	0.127
GFAP	3NT	AMY	DFP+SAR	0.064

Table S4. Summary of Regression for Glial cell markers and $GP91^{phox}$. Indicates p<0.05

Glial cell marker	Oxidative stress marker	Region	Experimental Group	R^2
IBA1	$GP91^{phox}$	PC	PBS+VEH	0.441
IBA1	$GP91^{phox}$	PC	PBS+SAR	0.022
IBA1	$GP91^{phox}$	PC	DFP+VEH	0.029
IBA1	$GP91^{phox}$	PC	DFP+SAR	0.178
IBA1	$GP91^{phox}$	AMY	PBS+VEH	0.196
IBA1	$GP91^{phox}$	AMY	PBS+SAR	0.020
IBA1	$GP91^{phox}$	AMY	DFP+VEH	0.002
IBA1	$GP91^{phox}$	AMY	DFP+SAR	**0.545***
GFAP	$GP91^{phox}$	PC	PBS+VEH	0.009
GFAP	$GP91^{phox}$	PC	PBS+SAR	0.020
GFAP	$GP91^{phox}$	PC	DFP+VEH	0.192
GFAP	$GP91^{phox}$	PC	DFP+SAR	0.010
GFAP	$GP91^{phox}$	AMY	PBS+VEH	0.007
GFAP	$GP91^{phox}$	AMY	PBS+SAR	0.132
GFAP	$GP91^{phox}$	AMY	DFP+VEH	0.058
GFAP	$GP91^{phox}$	AMY	DFP+SAR	0.352
