# Supplementary figures and images for: Saracatinib, a Src Tyrosine Kinase Inhibitor, as a Disease Modifier in the Rat DFP Model: Sex Differences, Neurobehavior, Gliosis, Neurodegeneration, and Nitro-Oxidative Stress
Source: Antioxidants (Basel). 2021 Dec 28;11(1):61. doi: 10.3390/antiox11010061 (PMC8773289; doi:10.3390/antiox11010061)

Figure S3

3NT original blot

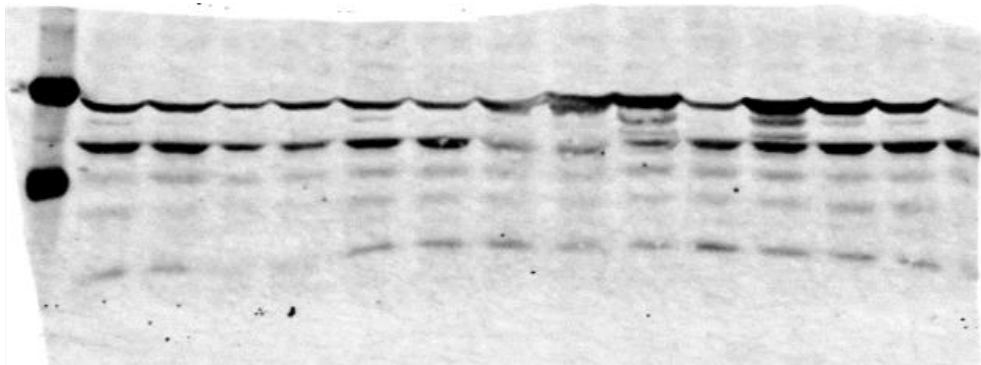

GP91 original blot

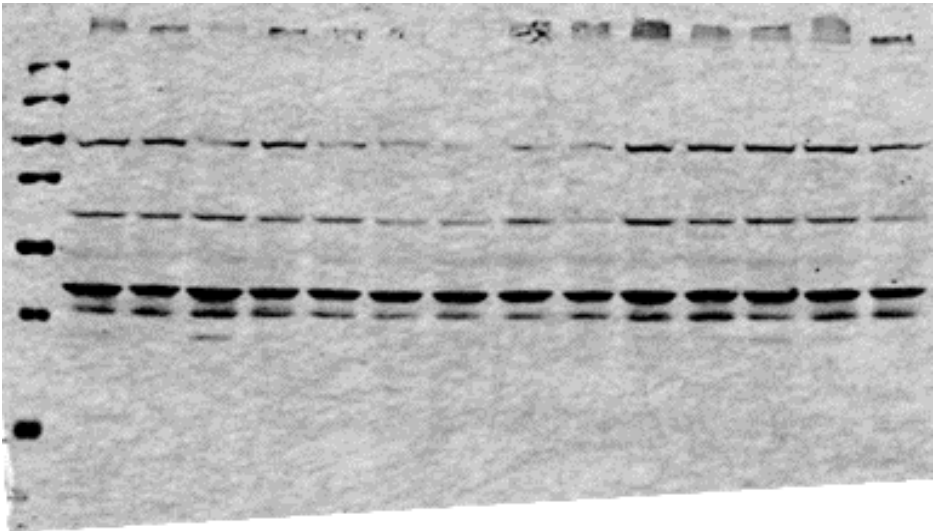

Supplement: Supplementary file 1 [file antioxidants-11-00061-s001.zip › Supplementary material/blots.pdf]
